# Contribution of antimicrobial photo-sonodynamic therapy in wound healing: an in vivo effect of curcumin-nisin-based poly (L-lactic acid) nanoparticle on *Acinetobacter baumannii* biofilms

**DOI:** 10.1186/s12866-022-02438-9

**Published:** 2022-01-17

**Authors:** Maryam Pourhajibagher, Babak Pourakbari, Abbas Bahador

**Affiliations:** 1 Dental Research Center, Dentistry Research Institute, Tehran University of Medical Sciences, Tehran, Iran; 2 Pediatric Infectious Disease Research Center, Tehran University of Medical Sciences, Tehran, Iran; 3 Pediatrics Center of Excellence, Children’s Medical Center, Tehran University of Medical Sciences, Tehran, Iran; 4 Department of Microbiology, School of Medicine, Tehran University of Medical Sciences, Tehran, Iran; 5 Fellowship in Clinical Laboratory Sciences, BioHealth Lab, Tehran, Iran

**Keywords:** Antimicrobial photodynamic therapy, Antimicrobial sonodynamic therapy, Biofilms, Burn wound infection, Curcumin, Nisin, Silver sulfadiazine

## Abstract

**Background:**

The biofilm-forming ability of *Acinetobacter baumannii* in the burn wound is clinically problematic due to the development of antibiotic-resistant characteristics, leading to new approaches for treatment being needed. In this study, antimicrobial photo-sonodynamic therapy (aPSDT) was used to assess the anti-biofilm efficacy and wound healing activity in mice with established *A. baumannii* infections.

**Methods:**

Following synthesis and confirmation of Curcumin-Nisin-based poly (L-lactic acid) nanoparticle (CurNisNp), its cytotoxic and release times were evaluated. After determination of the sub-significant reduction (SSR) doses of CurNisNp, irradiation time of light, and ultrasound intensity against *A. baumannii*, anti-biofilm activity and the intracellular reactive oxygen species (ROS) generation were evaluated. The antibacterial and anti-virulence effects, as well as, histopathological examination of the burn wound sites of treated mice by CurNisNp-mediated aPSDT^SSR^ were assessed and compared with silver sulfadiazine (SSD) as the standard treatment group.

**Results:**

The results showed that non-cytotoxic CurNisNp has a homogeneous surface and a sphere-shaped vesicle with continuous release until the 14th day. The dose-dependent reduction in cell viability of *A. baumannii* was achieved by increasing the concentrations of CurNisNp, irradiation time of light, and ultrasound intensity. There was a time-dependent reduction in biofilm growth, changes in gene expression, and promotion in wound healing by the acceleration of skin re-epithelialization in mice. Not only there was no significant difference between aPSDT^SSR^ and SSD groups in antibacterial and anti-virulence activities, but also wound healing and re-epithelialization occurred more efficiently in aPSDT^SSR^ than in the SSD group.

**Conclusions:**

In conclusion, CurNisNp-mediated aPSDT might be a promising complementary approach to treat burn wound infections.

## Background

Burn wound infection is one of the common and serious healthcare problems [1]. Many factors such as type, amount of the microbial burden colonizing in burn wounds, as well as, the ability of microbial biofilm formation are the risk factors in morbidity and mortality in burn patients [2]. Burn wounds are colonized by Gram-positive bacteria derived from skin commensals, followed later by Gram-negative bacteria and yeasts [3]. *Acinetobacter baumannii* is an important opportunistic bacterium capable of developing a wide range of infections in burn wounds [4]. It emerged as the common and invasive bacteria under its robust antibacterial resistance and virulence factor. The inappropriate over use of antibiotics in empirical therapy led to multidrug-resistant *A. baumannii* outbreaks all over the world [5, 6]. Therefore, a key challenge is to find new non-antibiotic therapies to eliminate *A. baumannii* from colonizing in burn wounds.

*A. baumannii* senses light through the photoreceptor protein “blue light sensing A”, which is encoded by the *blsA* gene. *blsA* expression prevents the formation of biofilm as one of the most important virulence factors and ultimately leads to increased susceptibility of bacteria to antimicrobial agents. The involvement of CsuE (*csuE*) and AbaI (*abaI*) proteins in the biofilm formation of *A. baumannii* is well-established. CsuE as one of the main *A. baumannii* adhesions involved in initial attachment as the first step for colonization and subsequent biofilm formation. AbaI, is an essential factor for production of the acyl-homoserine lactone signal, which plays critical role in biofilm development. The loss of functional *csuE* and *abaI* activities in *A. baumannii* are corresponding decrease in growth and biofilm formation rate in soft-tissue infection [7].

Recently, it was reported that curcumin-nisin (CurNis) formulation is a non-toxic compound with antimicrobial and anti-inflammatory activities [8]. Nisin as an antimicrobial peptide can be used synergistically in combination with conventional therapeutic agents and activate the adaptive immune response and have an immunomodulatory role. Also, the potential for using nisin to treat local site-specific infections such as skin infections has been reported [9]. Curcumin, a natural polyphenol compound derived from the turmeric rhizomes (*Curcuma longa* L.), through its polyphenol nature and the presence of methoxy and hydroxyl groups, is attributed to many properties, in particular antioxidant, anti-inflammatory, antimicrobial, antimutagenic, and anti-angiogenic ones [10, 11]. The great potential effects of curcumin against chronic diseases including irritable bowel syndrome (IBS) and renal protection from high doses of naproxen in the rat model are due to its antioxidant and anti-inflammatory activities [12]. There is one meta-analysis based on randomized clinical trials, performed by Jakubczyk et al. [11, 12], describing an intervention of pure curcumin, which increased total antioxidant capacity (SMD = 2.696, Z = 2.003, CI = 95%, *p* = 0.045). Despite the therapeutic uses of these compounds, they have a short half-life, poor pharmacokinetics, poor bioavailability, low solubility, and instability, which limits their therapeutic effect in vivo [13]. Therefore, using a biodegradable polymer such as poly (L-lactic acid) for the development of nanocarrier can promote their effective therapeutic usage [14].

Studies also showed that Cur has significant antimicrobial effects at very low molarity following activation by light and/or ultrasound in the process of antimicrobial photodynamic therapy (aPDT) and antimicrobial sonodynamic therapy (aSDT) [13, 15–19]. aPDT is a potential alternative approach, which is the photoinactivation of microorganisms and thereby kill cells by reactive oxygen species (ROS) generated by a harmless visible light-activated nontoxic photosensitizer in the presence of oxygen. According to the literature, aPDT has antimicrobial effects on bacteria isolated from infected human burn wounds and improves wound healing in mice and humans [20–23]. Sun et al. reported aPDT may be an appropriate alternative to standard therapies for infected wounds [24]. aSDT, as a novel ultrasound-driven treatment has been found to be very effective in killing microorganisms due to its strong penetrating power through a sonochemical process [17]. Several recent reports have mentioned satisfactory results with aSDT in inhibiting microorganisms due to its non-invasive nature [17, 18, 25]. The main advantage of aSDT over aPDT is the increased penetration of ultrasound irradiation to the target site compared to light [17]. aPDT and aSDT are the local therapies and can reduce side effects of systemic administration of antimicrobial agents. Another advance in aPDT and aSDT is the management of multidrug-resistant bacterial infections and the produced ROS by them cause broad-spectrum oxidative damage to the target cells Interestingly, aPDT and aSDT are promising strategies to which to date no resistant strain has been reported [18, 25].

It has been shown that silver sulfadiazine (SSD) is an effective topical antibiotic for treatment and protect of second-degree (partial-thickness) and third-degree (full-thickness) burns, respectively, which should be apply for several consecutive days [26]. Along with the beneficial antimicrobial properties of SSD, its major disadvantage is release of silver (Ag+) ions in high doses and the toxic effects on keratinocytes and fibroblasts [27]. Due to the increasing need for the effective treatment of burn wound infections, more attention in this study has been focused on the simultaneous use of aPDT and aSDT, which is called antimicrobial photo-sonodynamic therapy (aPSDT). Herein, we explored the anti-biofilm efficacy of aPSDT using CurNis-based poly (L-lactic acid) nanoparticles (CurNisNp) as a photo-sonosensitizer to eliminate the biofilms of *A. baumannii* on surfaces of wounds in the animal model. It was hypothesized that CurNisNp-mediated aPSDT will remove the *A. baumannii* biofilms and improve wound healing more than aPDT and/or aSDT and provide an experimental basis for future clinical application in the treatment of burn wound infections.

## Results

### Confirmation of synthesized CurNisNp

**Fig. 1:**
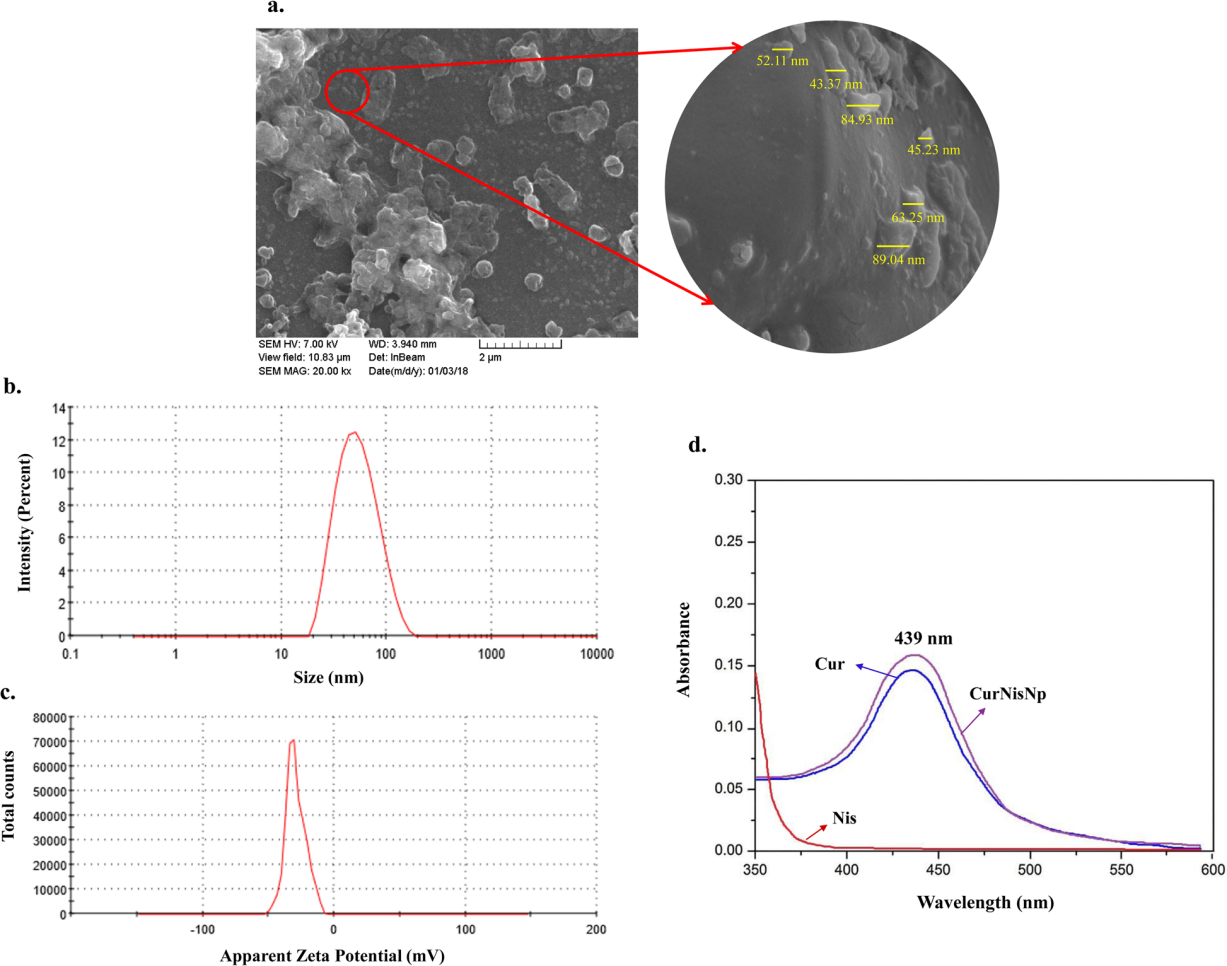
Characterization of synthesized Curcumin-Nisin-based poly (L-lactic acid) nanoparticle (CurNisNp): **a**) Field emission scanning electron microscope (FESEM) image (Scale bar = 2 μm), **b**) Average diameter, **c**) Zeta potential, and **d**) Ultraviolet–Visible spectra. Abbreviation; Nis: Nisin, Cur: Curcumin

Surface morphology of synthesized CurNis in nano scale was confirmed using FESEM analysis. As shown in Fig. 1a, the surface of CurNisNp appeared to be homogeneous with a spherical shape indicating acceptable compatibility between Cur-Nis and poly (L-lactic acid). The average diameter, PDI, and zeta potential of CurNisNp were approximately 78.6 ± 17.9 nm, 0.171 ± 0.04, and − 30.7 ± 4.84 mV, respectively (Fig. 1b, c). Ultraviolet-Visible (UV–Vis) spectra of pure Cur, Nis, and CurNisNp are presented in Fig. 1d. Accordingly, the absorption spectra of synthesized CurNisNp showed an absorbance peak at 439 nm.

### In vitro drug release

**Fig. 2:**
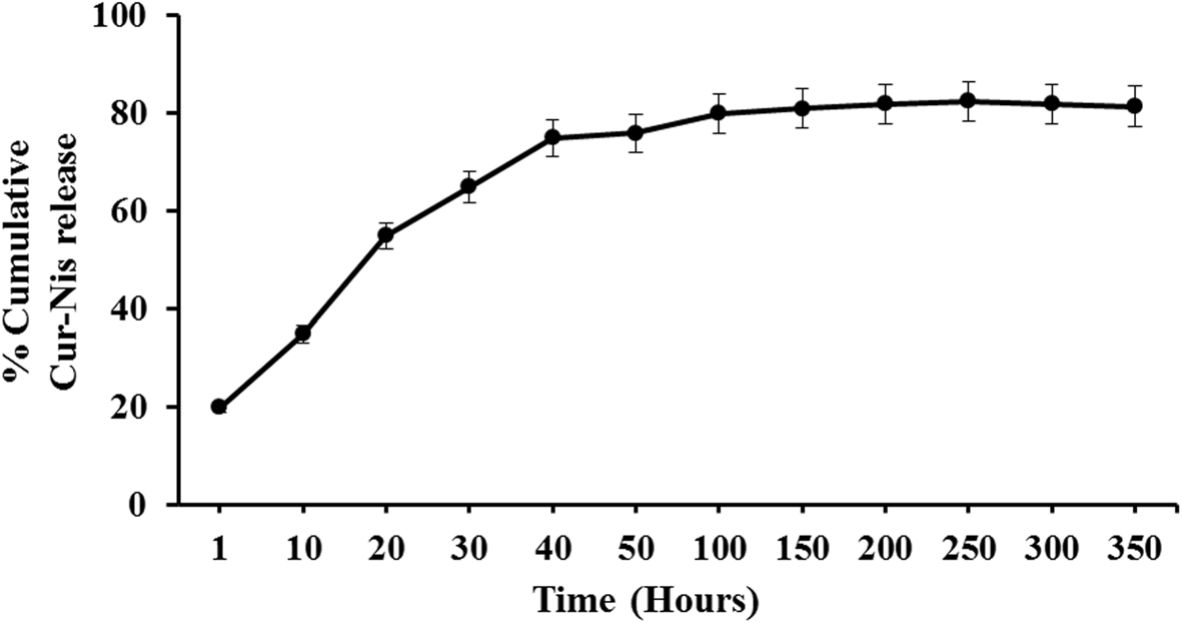
In vitro drug release of Curcumin-Nisin (Cur-Nis) from Curcumin-Nisin-based poly (L-lactic acid) nanoparticle (CurNisNp) during 14 days

Standard plot obtained from UV-absorbance analysis of free Cur-Nis was used to estimate from the release kinetics of CurNis-entrapped nanoparticle*.* In vitro release of CurNis from CurNisNp showed a rapid initial burst at the first hour followed by a sustained release for 14 days. Around 20–40% of the total entrapped CurNis was released within 10 h and around 80% was released at 4 days. This in vitro release profile showed that continuous release of CurNis was continued until the 14th day (Fig. 2).

### Cytotoxicity assessment of CurNisNp on normal human skin fibroblast cell line (MHFB-1) cells

**Fig. 3:**
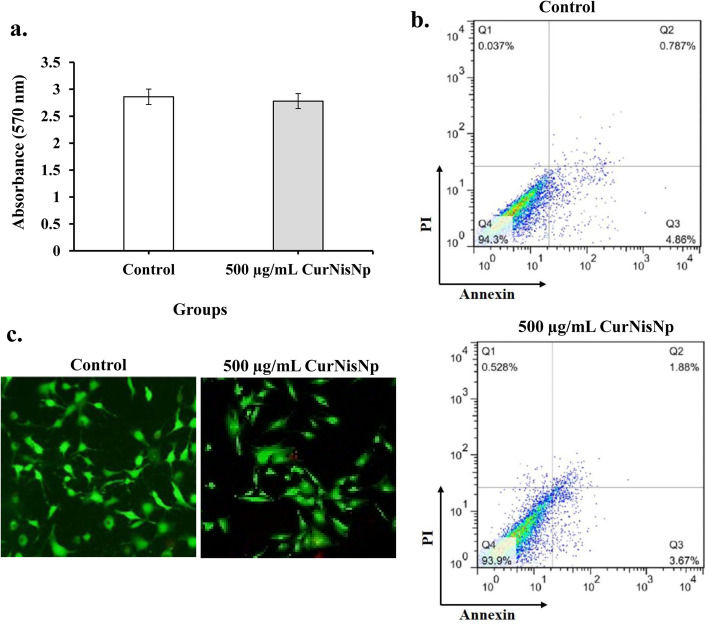
Cytotoxic effects of Curcumin-Nisin-based poly (L-lactic acid) nanoparticle (CurNisNp) on cell viability of normal human skin fibroblast cell line (MHFB-1): **a**) MTT assay, **b**) Annexin V-FITC/Propidium iodide (PI) staining detected by flow cytometry, and **c**) Fluorescent micrograph of acridine orange (AO) and ethidium bromide (EB) staining of cells

Cytotoxicity analysis using MTT assay kit demonstrated that CurNisNp at the highest concentration (500 μg/mL) was not significantly toxic to MHFB-1 (Fig. 3a). In the flow cytometry assay shown in Fig. 3b, the cell apoptosis rate was very negligible. Acridine orange (AO) and ethidium bromide (EB) fluorescent staining is a method to analyze the viable cells, early apoptosis, and late apoptosis, as well as the morphologic changes in cells. As the results shown, untreated and treated MHFB-1 cells showed viable cells with green color and intact nuclei (Fig. 3c). Taken together, these data demonstrate that CurNisNp had no cytotoxic effect on cells.

### SSR doses of CurNisNp, irradiation time of LED, and ultrasound intensity against *A. baumannii*

**Fig. 4:**
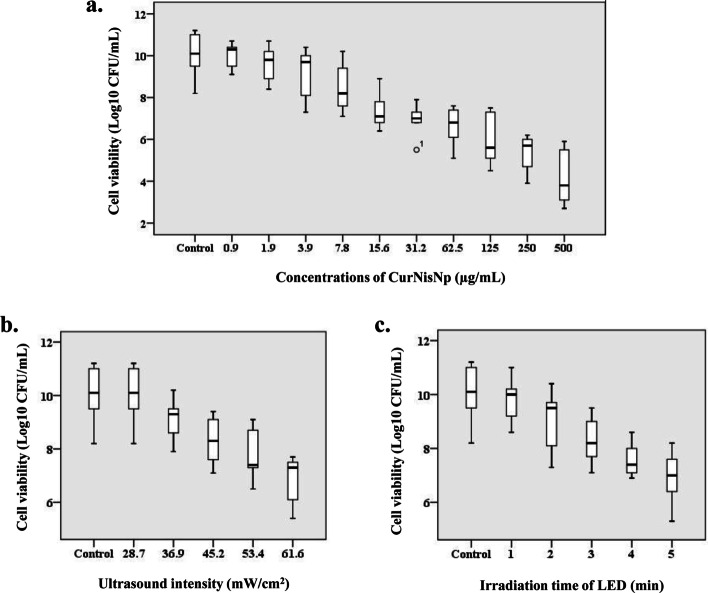
Cell viability of *A. baumannii* in different treatment groups: **a**) Curcumin-Nisin-based poly (L-lactic acid) nanoparticle (CurNisNp), **b**) Ultrasound intensity, and **c**) Irradiation time of light emitting diode (LED)

The microbroth dilution assay were performed to detect the SSR dose of CurNisNp (CurNisNp^SSR^) against *A. baumannii*. The data indicate that the dose-dependent reduction in cell viability was induced by increasing the concentrations of CurNisNp. When 31.2 to 500 μg/mL CurNisNp were used, the cell viability of *A. baumannii* significantly decreased compared to the control group (*P* < 0.05), whereas there was no remarkable reduction when CurNisNp was decreased from 15.6 to 0.9 μg/mL (*P* > 0.05). So, the maximum CurNisNp^SSR^ against *A. baumannii* was found to be 15.6 μg/mL with the median (interquartile range [IQR]) log_10_ CFU/mL of 7.1 (6.8–8.9) (Fig. 4a).

Also, the cell viability of *A. baumannii* was decreased with the enhancement of irradiation time of LED and irradiation intensity of ultrasound waves. The log_10_ CFU/mL reductions in cell viability of *A. baumannii* were demonstrated at ultrasound intensity of 53.4 and 61.6 mW/cm^2^, respectively (*P* < 0.05). Additionally, there was a significant decrease in *A. baumannii* cell viability following exposure to LED with the energy density of 300–420 J/cm^2^ for 5 min (P < 0.05). Taking into account the cell survival rate, the maximum SSR doses of irradiation time of LED (LED^SSR^) and irradiation intensity of ultrasound waves (US^SSR^) were 4 min (energy density of 252–336 J/cm^2^) and 45.2 mW/cm^2^, respectively (*P* > 0.05) with the median (IQR) log_10_ CFU/mL of 7.4 (6.9–8.6) and 8.3 (7.1–9.4), respectively (Fig. 4b, c).

### Antimicrobial effects of CurNisNp, LED irradiation, ultrasound waves, and their combination against *A. baumannii* in planktonic growth

**Fig. 5:**
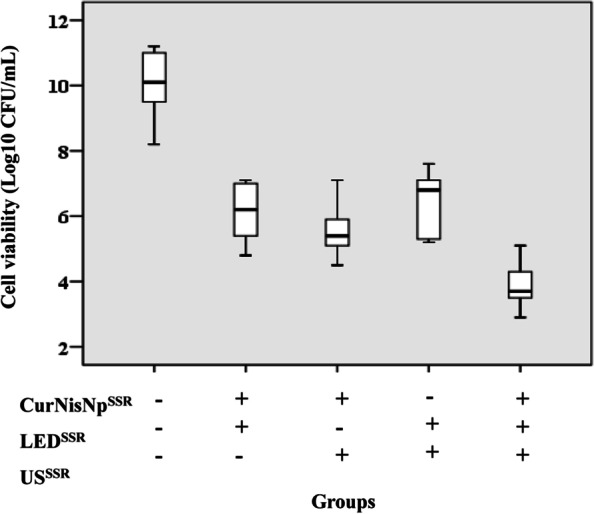
Cell viability of *A. baumannii* following treatment with sub-significant reduction doses of Curcumin-Nisin-based poly (L-lactic acid) nanoparticle (CurNisNp^SSR^), ultrasound intensity (US^SSR^), and Irradiation time of light emitting diode (LED^SSR^)

The results in Fig. 5 showed that all treatment groups could decrease the cell viability of *A. baumannii* in planktonic growth compared with the control group (*P* < 0.05). As shown in Fig. 5, the median (IQR) log_10_ CFU/mL of CurNisNp^SSR^ plus LED^SSR^, CurNisNp^SSR^ plus US^SSR^, LED^SSR^ plus LED^SSR^, and CurNisNp^SSR^ plus LED^SSR^ plus US^SSR^ were 6.2 (4.8–7.1), 5.4 (4.5–7.1), 6.8 (5.2–7.6), and 3.7 (2.9–5.1), respectively.

### Anti-biofilm effects of treatment groups against *A. baumannii*

Anti-biofilm activities of different treatment groups against *A. baumannii* were determined using colorimetric assay. The anti-biofilm activity of different treatment groups was evaluated by colorimetric assay. The mean ± standard deviation (SD) and median (IQR) of OD value of *A. baumannii* biofilm following the different treatments are presented in Table 1. All the aPDT^SSR^, aSDT^SSR^, and aPSDT^SSR^ groups based on CurNisNp had anti-biofilm effects against *A. baumannii* and could considerably reduce the bacterial counts in biofilm structures in comparison with the control group (*P* < 0.05). CurNisNp-aPSDT^SSR^ showed a significantly higher anti-biofilm activity than the other treatment groups (P < 0.05). Although the biofilms of *A. baumannii* are displayed to be more susceptible to CurNisNp-aSDT^SSR^ than CurNisNp-aPDT^SSR^, this difference is not significant (*P* > 0.05). According to the results in Table 1, although there is no significant difference in the reduction of biofilm between the SSD group with CurNisNp-aPDT^SSR^ (*P* = 0.129) and CurNisNp-aSDT^SSR^ (*P* = 0.968), but CurNisNp-aPSDT^SSR^ compared to SSD was able to significantly reduce the *A. baumannii* biofilm (*P* = 0.001).

**Table 1:** Anti-biofilm effects of different treatment groups against *A. baumannii* biofilms

Groups	Mean ± SD of OD at 570 nm	Median (IQR) of OD at 570 nm	Reduction of OD (%)	***P*** value
**Control**	3.26 ± 0.088	3.28 (3.15–3.36)	–	–
**LED**^**SSR**^	2.76 ± 0.144	2.73 (2.61–2.93)	15.3	0.000
**US**^**SSR**^	2.60 ± 0.070	2.57 (2.53–2.70)	20.2	0.000
**CurNisNp**^**SSR**^	2.52 ± 0.066	2.53 (2.43–2.61)	22.5	0.000
**CurNisNp-aPDT**^**SSR**^	0.93 ± 0.079	0.91 (0.85–1.05)	71.4	0.000
**SSD**	0.80 ± 0.141	0.76 (0.65–1.00)	75.3	0.000
**CurNisNp- aSDT**^**SSR**^	0.75 ± 0.105	0.78 (0.68–0.96)	76.8	0.000
**CurNisNp-aPSDT**^**SSR**^	0.20 ± 0.058	0.21 (0.13–0.26)	93.6	0.000

### Intracellular ROS generation

To investigate whether ROS is involved in aPDT and aSDT-induced antimicrobial effect, the levels of intracellular ROS generation in treated *A. baumannii* cells were assessed. *A. baumannii* cells when incubated in presence in CurNisNp alone and SSD, showed no remarkable ROS generation (2.3-fold and 0.2-fold, respectively; *P* > 0.05), while, a significant ROS production was observed when cells were treated by CurNisNp and irradiated with ultrasound waves (13.7-fold; *P* < 0.05) and LED (15.2-fold; P < 0.05). Also, no considerable ROS generation was observed when cells were exposed to aPDT and aSDT (P > 0.05).

### In vivo assessment of wound healing

**Fig. 6:**
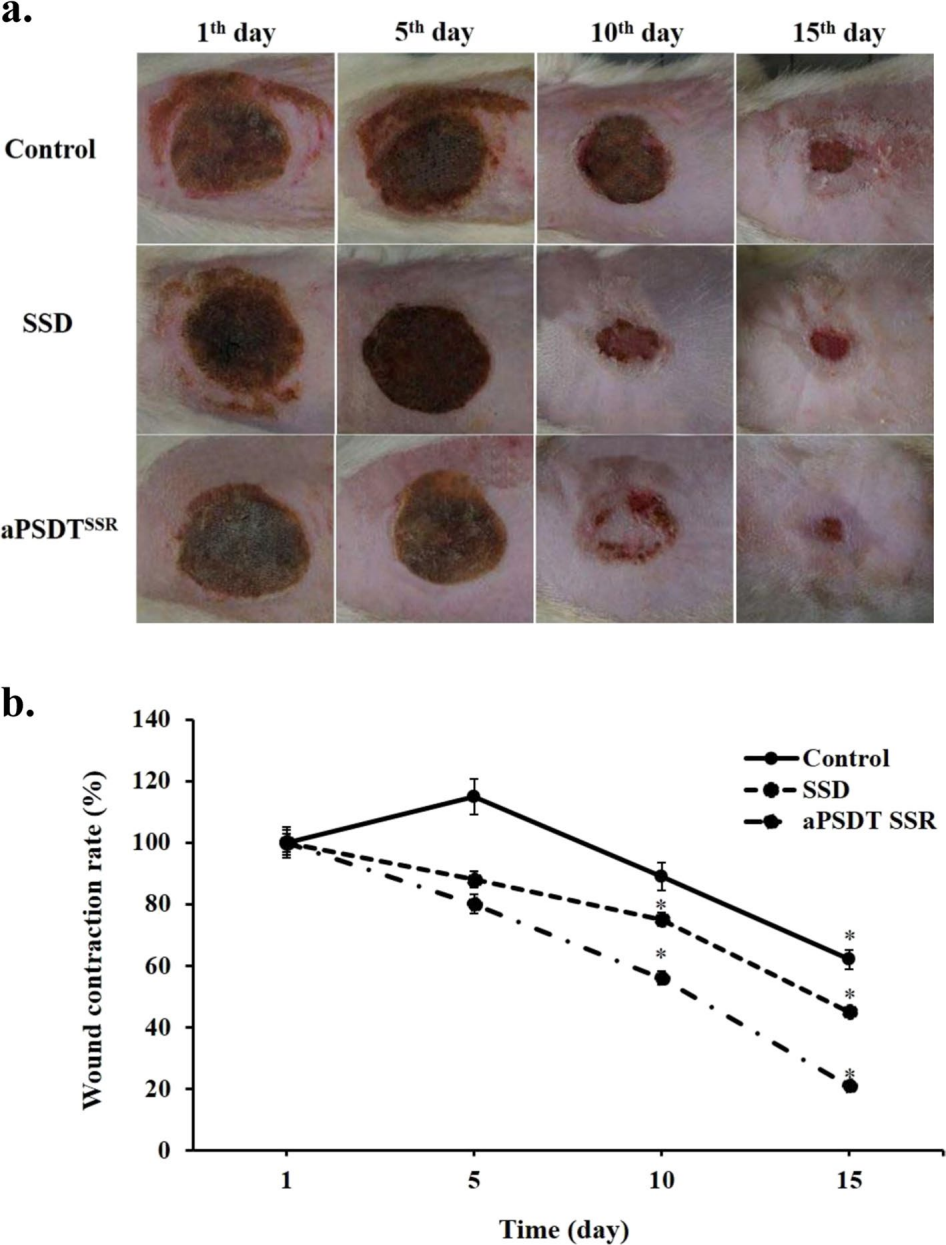
Wound healing process observation: **a**) Serial wound healing process on day 1, day 5, day 10, and day 15 under gross observation in CurNisNp at sub-significant reduction dose + Irradiation time of light emitting diode at sub-significant reduction dose + Ultrasound intensity at sub-significant reduction dose (aPSDT^SSR^), Silver sulfadiazine (SSD), and no treatment (Control) groups, **b**) Changes in treated scald wound sizes with CurNisNp at sub-significant reduction dose + Irradiation time of light source at sub-significant reduction dose + Ultrasound intensity at sub-significant reduction dose (aPSDT^SSR^), Silver sulfadiazine (SSD), and no treatment (Control) groups on day 1, day 5, day 10, and day 15. ∗P < 0.05

Wound healing process and the percentage of wound contracture rate was shown in Fig. 6. By day 5, all groups showed thick scabs. After day 10, wound healing was accelerated with application of aPSDT^SSR^ compared to SSD and control groups. As shown in Fig. 6, aPSDT^SSR^ significantly enhanced wound closure and re-epithelialization during burn wound healing process (*P* < 0.05). On day 14, the wound surfaces were almost closed and wound sizes were smallest in aPSDT^SSR^ groups.

### In vivo antibacterial effects of CurNisNp-mediated aPSDT on infections of burn wounds

**Fig. 7:**
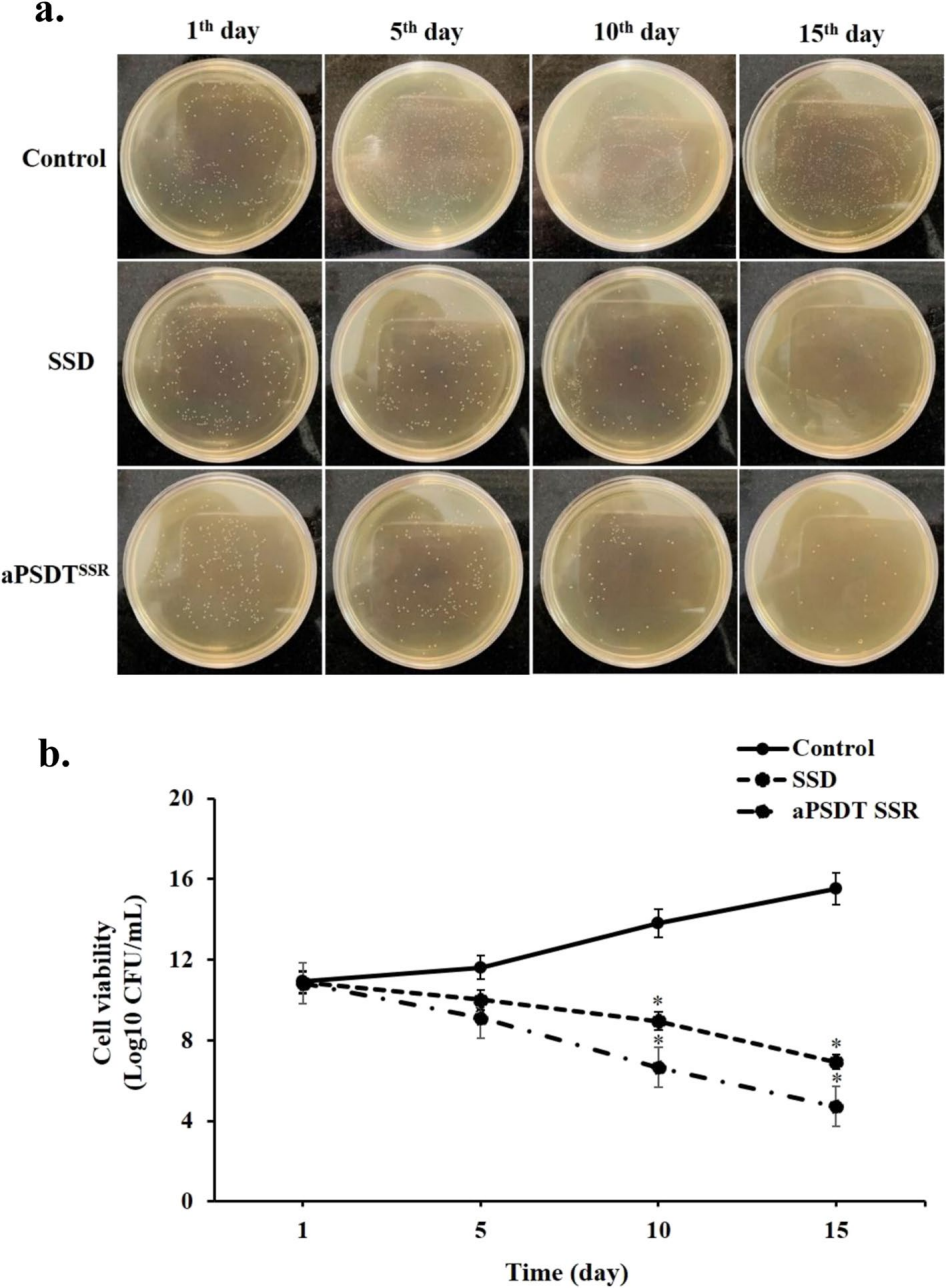
In vivo antibacterial effects of CurNisNp at sub-significant reduction dose + Irradiation time of light emitting diode at sub-significant reduction dose + Ultrasound intensity at sub-significant reduction dose (aPSDT^SSR^), Silver sulfadiazine (SSD) and no treatment (Control) groups against *A. baumannii* on burn wound: a) Bacterial colonies on the agar plates, b) Log_10_ colony forming unit (CFU)/mL counts of *A. baumannii.* ∗*P* < 0.05

We used mouse, a well-known burn wound model, to explore the antibacterial effects of CurNisNp-mediated aPSDT. In this study, the antibacterial effects of aPSDT^SSR^ and SSD groups were studied on burn wound infections in a mouse model. The results show that the time-dependent reduction in cell viability was induced with the passing of the days. According to the results in Fig. 7, at the 5th, 10th, and 15th days, successful antibacterial effects against treated *A. baumannii* by aPSDT^SSR^ and SSD were achieved (*P* < 0.05). aPSDT^SSR^ group could reduce the amount of *A. baumannii* by up to 4.72 ± 0.15 log_10_ CFU/mL compared with the no treatment group on the 15th day (P < 0.05)*,* while SSD was able to reduce the amount of bacteria by up to 6.93 ± 1.30 log_10_ CFU/mL in comparison with the control group (*P* < 0.05).

### Effects of CurNisNp-mediated aPSDT on virulence gene expression patterns in *A. baumannii*

**Fig. 8:**
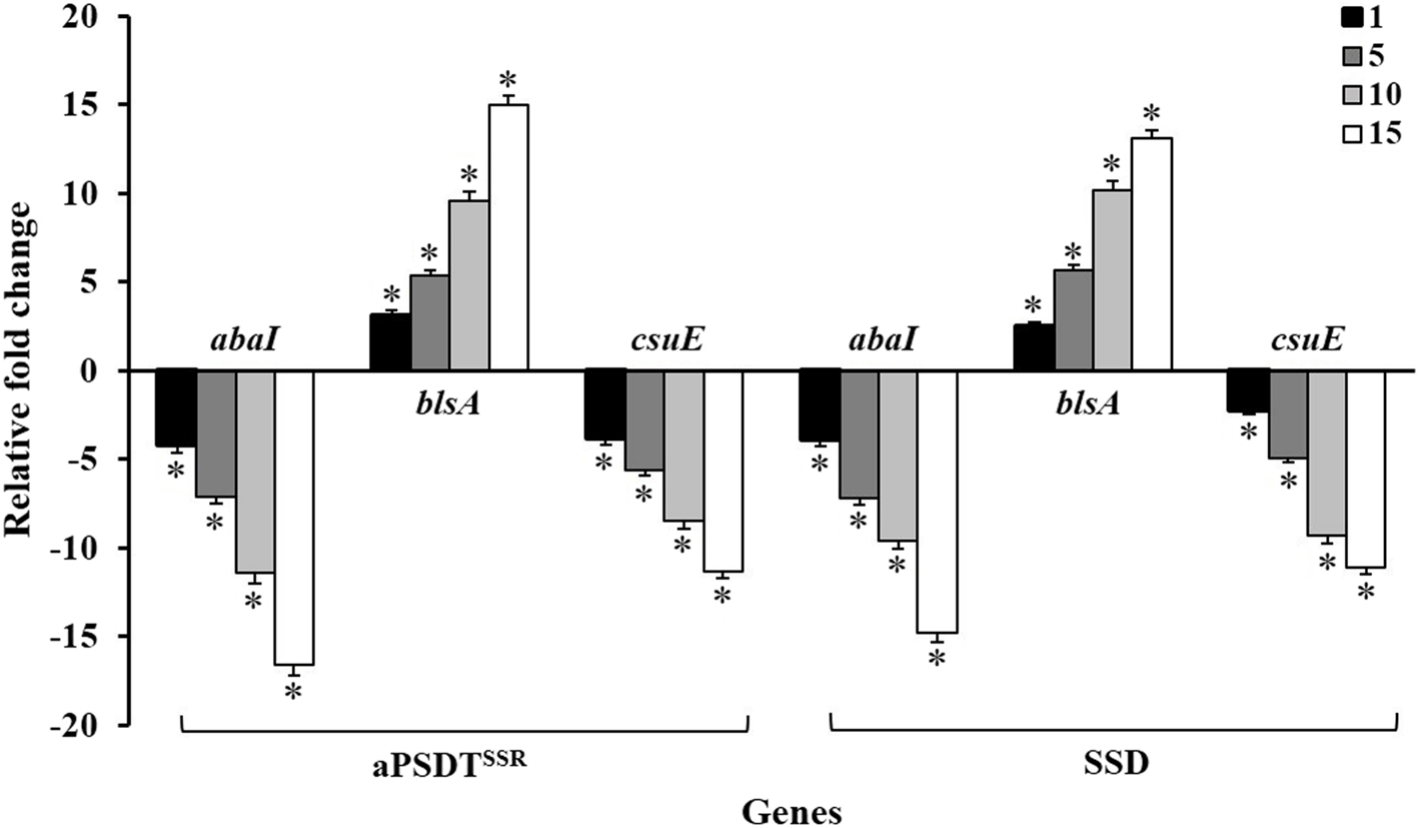
Relative fold change in mRNA expression level of virulence genes on days 1, 5, 10, and 15 in *A. baumannii* treated with CurNisNp at sub-significant reduction dose + Irradiation time of light emitting diode at sub-significant reduction dose + Ultrasound intensity at sub-significant reduction dose (aPSDT^SSR^) and Silver sulfadiazine (SSD) groups. ∗P < 0.05

To explore the mechanism underlying the anti-virulence induced by CurNisNp-mediated aPSDT, the gene expression patterns in *A. baumannii* was determined using quantitative real-time PCR. After treatment of *A. baumannii* using aPSDT^SSR^ and SSD groups, the changes in the expression of virulence genes were evaluated at various time intervals. As shown in Fig. 8, changes in gene expression are time-dependent. In aPSDT^SSR^-treated *A. baumannii*, mRNA expression was considerably upregulated to 3.1-, 5.4-, 9.6-, and 15.0-folds in *blsA* on the 1st, 5th, 10th, and 15th days, respectively (*P* < 0.05). In contrast, the expression of genes involved in the formation of microbial biofilm (*abaI* and *csuE*) was significantly downregulated (P < 0.05). Also, the change in gene expression in the SSD group was similar to aPSDT^SSR^ and no significant difference was observed between them (*P* > 0.05). According to the results, a significant reduction in *abaI* and *csuE* expression and a considerable increase in *blsA* expression was observed on all test days (P < 0.05).

### Histopathological analysis

**Fig. 9:**
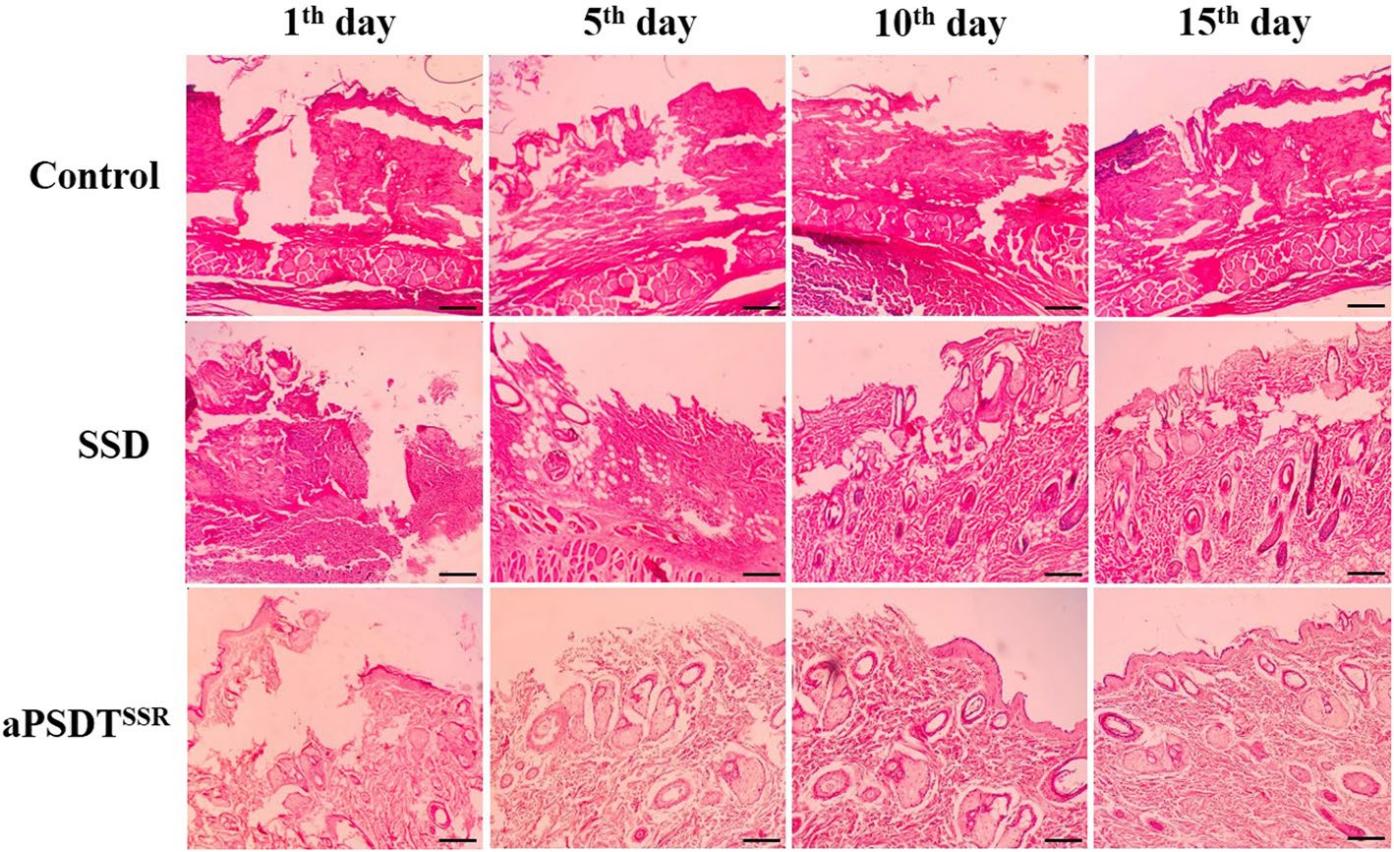
Photomicrographs of tissue sections stained with hematoxylin-eosin (HE) on the days 1, 5, 10, and 15 after treatment with CurNisNp at sub-significant reduction dose + Irradiation time of light emitting diode at sub-significant reduction dose + Ultrasound intensity at sub-significant reduction dose (aPSDT^SSR^), Silver sulfadiazine (SSD) and no treatment (Control) groups (10X mag; scale bar represents 0.1 mm)

To investigate whether CurNisNp are involved in aPSDT^SSR^-induced wound healing, the evaluation of inflammation, fibroblasts, blood vessels, and re-epithelialization in cross-sectioned tissue obtained from each treated burn wound were assessed by the hematoxylin-eosin (HE) staining under a general optical microscope. Histologically, the dermal inflammatory cell infiltration, abscess formation, absence of collagen distinction, and loss of epidermis were observed in the wounds on 1st day (Fig. 9). The proliferation of marginal epithelium of ulcer was initiated on the 5th day post aPSDT^SSR^ and SSD. On 10th day, continuing re-epithelialization was observed in both aPSDT^SSR^ and SSD groups compared to the control group. In addition, dermal closure and granulation-tissue formation were initiated. Complete tissue re-epithelialization, fibroblastic proliferation, presence of modeled dense collagen mesh, and moderate fibrosis were the important findings on the 15th day. Day 15 observations suggest that the wound healing effect of aPSDT^SSR^ is related to the activation of granulation tissue formation, as well as, collagen regeneration occurred more efficiently in aPSDT^SSR^ than in the SSD group.

## Discussion

aSDT is analogous to aPDT except that drug activation is obtained via ultrasound instead of light [17]. Similar to aPDT, aSDT process generates ROS through the ultrasound-mediated stimulated sonosensitizer in the presence of O_2_ and the ultrasound-activated cavitation effects can destruct the microbial cells [15, 17]. Following the development of the biological activity of aPDT and aSDT in recent years, many sensitizers were found to have both photodynamic and sonodynamic antimicrobial effects. Therefore, aPSDT has been taken into consideration to obtain better treatment outcomes by reducing the dose of both light energy/ultrasound irradiation and sensitizers, in resulting reducing the cytotoxicity effects [28].

An appropriate photo-sonosensitizer should have low toxicity to the host cells and high toxicity to the target cells when associated with ultrasound irradiation and/or illumination. The results of the current study showed that there was negligible cytotoxicity against MHFB-1 cells, suggesting that the synthesized CurNisNp had the least toxicity against eukaryotic cells and good biocompatibility with the host cells.

Recently, the photo-sono-biological and photo-sono-killing potential of Cur has been explored due to its capability of producing ROS, a free radical, and can play a role in intracellular compounds, activating transcription factors, and altering cytokines [15–19]. In this study, poly (L-lactic acid) as a nanocarrier was used to overcome the poor bioavailability of Cur-Nis. poly (L-lactic acid) is one of the most successfully used biodegradable polymers for drug delivery system and produce the non-toxic biodegradable metabolite monomers following its hydrolysis within the body [29]. The obtained CurNisNp was well-dispersed in an aqueous solution with a narrow particle size distribution. Additionally, when Cur-Nis is encapsulated in the poly (L-lactic acid), it is segregated from the aqueous phase and can release continuously until 2 weeks [8, 30].

Biofilm-related infections are significantly resistant to clearance by the host immune system and various antimicrobial agents [31]. It is evident that biofilm-forming ability can be considered one of the main virulence factors of *A. baumannii*. Biofilm-producing *A. baumannii* strains manifest an altered growth rate and transcribe genes that provide them with inherent resistance to living in strict environments, routine antibiotics, and the host immune system [4, 32].

The biofilm killing/degradation in vitro was assessed with the colorimetric method based on crystal violate assay. A significant reduction was observed at CurNisNp-mediated aPDT^SSR^ (71.4%), aSDT^SSR^ (76.8%), and aPSDT^SSR^ (93.6%) when were compared with the control group.

The objective of this study was to determine whether ultrasound irradiation in aSDT can be used to enhance CurNisNp-mediated aPDT to eliminate the microbial biofilms of *A. baumannii* in vitro and in vivo. According to the literature, no studies have been published regarding the concurrent application of aPSDT in eradicating *A. baumannii* biofilms. Although a limited number of studies have evaluated the effect of aPSDT on other microorganisms, their results are consistent with the results of this study [33–36].

The results of Pourhajibagher et al. [33] showed that aPSDT could provide a means of circumventing the limitations of decontamination of the dental implant surfaces. In their ex vivo study, chitosan nanoparticles indocyanine green-mediated aPSDT decreased the polymicrobial periopathogenic biofilms on surfaces of the titanium dental implants to 90.5%. The inactivation of *Candida albicans* biofilms using combined aPDT/aSDT in oropharyngeal candidiasis was highlighted by Alves et al. [18]. Xu et al. [37] reported that synergistic aPDT and aSDT improved the inhibition rate of antibiotic-resistant bacteria in infectious diseases compared with either the aPDT or aSDT alone.

During the aPSDT, phot-sonosensitizer attaches to the surface of microbial cells, penetration into the cells, and will be activated by relatively low-intensity ultrasound and visible light irradiation. When phot-sonosensitizer-treated microbial cells are received the ultrasound waves in aqueous micro-environments, result in microbubbles and cavitation which implosive collapse of gas-filled microbubbles. After that, released energy can be transferred to the oxygen and generate ROS. On the other hand, the excited photo-sonosensitizer reacts with biomolecules through electron transfer to form radicals, which react with oxygen to generate ROS (Type I); and/or react directly with oxygen through energy transfer, generating singlet oxygen (Type II). The present data provide indirect evidence that one of the mechanisms of aPSDT is probably based on induction of ROS production and this will result in target-cell death [4, 36, 38–40].

Several studies confirmed that the intracellular ROS production was greatly increased in aPDT and/or aSDT compared with the ultrasound, light, and photosensitizer/or sonosensitizer alone [41–45]. We evaluated intracellular ROS generation by DCFH-DA fluorescence and showed revealed CurNisNp can be activated using both ultrasound and light to produce more ROS for aPSDT against *A. baumannii* biofilms. Moreover, ROS generation was greatly increased in aPSDT^SSR^ compared with aPDT^SSR^ and aSDT^SSR^.

The relationship between biofilm formation and related genes was evaluated previously [46]. In this study, we also compared the levels of virulence-associated genes expression such as *blsA, abaI,* and *csuE* in treated *A. baumannii* by aPDT^SSR^, aSDT^SS^R, and aPSDT^SSR^. The evidence obtained suggests that *blsA* plays a positive role in the virulence of *A. baumannii* due to its global effect on *A. baumannii* physiology and results finally in increased sensitivity of bacteria to the antimicrobial agents [47]. The production of pili which is required for the initial steps of biofilm formation and development of *A. baumannii*, is intermediated by the *csuE* [48]. Also, AbaI, encoded by *abaI*, as a quorum-sensing molecule that catalyzes the synthesis of AHL signals, is a required factor for the production of biofilm on abiotic surfaces [49]. In this study, there was a significant downregulation in *abaI* and *csuE* expression, as well as a considerable upregulation in *blsA* expression in *A. baumannii* during aPSDT^SSR^ in comparison with the other groups. On the other hand, the findings show that changes in gene expression were time-dependent. On the 15th day, the highest and lowest changes in gene expression were reported. Our results demonstrated that CurNisNp-aPSDT^SSR^ could kill bacteria and eliminate *A. baumannii* biofilms, as well as, promote wound healing in mice. Additionally, not only there was no significant difference between CurNisNp-aPSDT^SSR^ and SSD groups in anti-biofilm and anti-virulence activities, but also wound healing and re-epithelialization occurred more efficiently in aPSDT^SSR^ than in the SSD group. Our findings warrant detailed examination of the interactions between the CurNisNp-aPSDT as an alternative therapy for the successful treatment of burn wound infections.

## Conclusion

In conclusion, this in vitro and in vivo study demonstrated that CurNisNp-aPSDT without any cytotoxicity on normal human skin fibroblast cell line could reduce the cell viability of *A. baumanni* via ROS generation. CurNisNp-aPSDT deceased the biofilm growth in *A. baumannii* by altering the expression of genes involved in bacterial pathogenesis and promote wound healing by the acceleration of skin re-epithelialization more than SSD as the standard treatment group. In addition, the results indicated that CurNisNp-aPSDT might be a promising complementary strategy to treat burn wound infections.

## Methods

### Synthesis of CurNisNp as the photo-sonosensitizer

CurNisNp was prepared by the double emulsion-diffusion-evaporation method [8]. Briefly, an equal amount of curcumin and nisin (5 mg) was dissolved in 200 μL 1% polyvinyl alcohol (PVA). 50 mg poly (L-lactic acid) was added to the compound and the mixture was then sonicated at 30 W for 1 min to form a primary emulsion. The emulsion was added dropwise to 16 mL 2% PVA and 1% sucrose. Following the sonication of nanosuspension at 30 W, 40% duty cycle for 3 min, the secondary emulsion was continuously stirred until all the solvents were evaporated. The nanosuspension was centrifuged (at 16,000×g for 15 min) and then washed three times. 5% mannitol was added to the nanosuspension and the formulation lyophilized to obtain the dry powder of CurNisNp.

### Characterization of CurNisNp

The surface morphology of CurNisNp was studied by field emission scanning electron microscopy (FESEM; ZEISS, German). The size distribution profiles of nanometer-sized particles, zeta potential, and polydispersity index (PDI) of the CurNisNp were carried out using a MALVERN Zetasizer Ver. 6.01 (Malvern Instruments, UK) at approximately 25 °C. Also, the absorption spectrum of CurNisNp was carried out by a UV-visible spectrophotometer, scanning the absorbance spectra in the range of 350–600 nm wavelength.

### In vitro Cur-Nis release study

In vitro release study of Cur-Nis from CurNisNp was performed as described by Omobhude et al. [30]. Briefly, 10 mg of CurNisNp was suspended in 10 ml PBS and a homogenous solution was kept on a rotatory shaker at 37 °C and 200×g. The samples were collected after centrifugation at 16,000×g for 15 min and the UV-absorbance analysis of supernatant was carried out at various time intervals.

### Screening of CurNisNp cytotoxicity by MTT assay, fluorescent staining, and flow cytometry analysis

The cellular cytotoxicity of the prepared CurNisNp was tested on normal human skin fibroblast cell line (MHFB-1; IBRC C11179) as described previously [50]. The cell was purchased from Iranian Biological Resource Center (Tehran, Iran) and grown in the Dulbecco’s Modified Eagle Medium/Nutrient Mixture F-12 (DMEM/F12) supplemented with 10% fetal bovine serum, 2 mM L-glutamine, 100 μg/mL amphotericin B and 1% penicillin/streptomycin antibiotic solution (all purchased from Sigma-Aldrich, Steinheim, Germany) in a humidified environment with 5% CO_2_ at 37 °C. MHFB-1 cells were seeded in 96-well microtiter plates at a density of 2.0 × 10^4^ cells per well. After 24 h, cells were washed with PBS and the highest concentration of CurNisNp (500 μg/mL) was added to triplicate wells and kept for 24 h at 37 °C, with 5% CO_2_/95% air in a humidified incubator. On one of the microtiter plates, a 3- (4,5-dimethylthiazol-2-yl)-2,5-diphenyltetrazolium bromide (MTT) assay kit (Sigma-Aldrich, Steinheim, Germany) was used to evaluate the viability of the cells with a microplate reader at 570 nm according to the manufacturer’s instructions. On another microtiter plate, the cells were resuspended in 100 μL binding buffer and incubated with 5 μL of Annexin V-FITC and propidium iodide (PI) as a counterstain for 15 min at room temperature, according to the manufacturer’s recommendations. Finally, percent of alive cells was evaluated by flow cytometry (Becton Dickinson) and data were analyzed using Flowjo software V7. For detection of alive cells by fluorescent staining, paraformaldehyde (4% in PBS)-fixed cells were stained with dual acridine orange (AO; 100 μg/mL)/ ethidium bromide (EB; 100 μg/ mL) fluorescent staining solution for 10 min as described previously [51]. The alive cells were assessed under the fluorescent microscope (OLYMPUS BX53, Japan).

### Light source

The light source used a light-emitting diode (LED, DY400–4, Denjoy, China) at the wavelength of 435 ± 10 nm with an output intensity of 1000–1400 mW/cm^2^. The medium temperature is stable at room temperature (25 ± 2 °C).

### Ultrasonic irradiation system

An ultrasound system was used as described previously [13]. The ultrasound parameters employed for treatment purposes were set as follows: a frequency of 1 MHz and pulsed repetition frequency of 100 Hz.

### Bacterial strain and growth conditions

*A. baumannii* ATCC 29212 strain obtained from Iranian Biological Resource Center has grown aerobically in brain heart infusion (BHI) broth (Laboratorios Conda S.A., Spain) at 37 °C. To examine the antimicrobial efficacy of CurNisNp- based aPSDT, *A. baumannii* suspension of approximately 1.5 × 10^8^ colony-forming unit (CFU)/mL was prepared using both spectrophotometry (optical density [OD] 600 nm: 0.08–0.13) and colony counting.

### Determination of antimicrobial effects of CurNisNp, LED irradiation, ultrasound waves, and their combination against *A. baumannii* in planktonic growth

The antimicrobial effects of CurNisNp, LED irradiation, ultrasound waves, and their combination, which would show as +/+/+, +/+/−, +/−/+, −/+/+, −/+/−, −/−/+, +/−/−, −/−/− were determined as follows:

1. *Determination of antimicrobial effects of CurNisNp against A. baumannii*

Antimicrobial effect of CurNisNp was determined according to the Clinical and Laboratory Standard Institute (CLSI) guidelines [52]. Briefly, 100 μL of CurNisNp at a final concentration of 500 μg/mL was diluted as two-fold serial dilutions with 100 μL of BHI broth in the wells of a 96-well microtiter plate. 100 μL/well of the *A. baumannii* (1.5 × 10^6^ CFU/mL) was added to each well and the microtiter plate was incubated at 37 °C. After 24 h, 10 μL of each well-containing dilution series were cultured onto BHI agar (Laboratorios Conda S.A., Spain) and log_10_ CFUs/mL were determined using the previous study [53]. The sub-significant reduction (SSR) dose of CurNisNp (CurNisNp^SSR^) was evaluated based on previous study [54].

2. *Determination of antimicrobial effects of LED against A. baumannii*

Antimicrobial effect of irradiation time of LED against *A. baumannii* was determined according to the previous study [54]. Briefly, 100 μL of *A. baumannii* suspension at the concentration of 1.5 × 10^5^ CFU/mL was added to the wells of a 96-well microtiter plate. *A. baumannii* cells were exposed to the LED at the wavelength of 435 ± 10 nm with an output intensity of 1000–1400 mW/cm^2^ for five variations of time irradiation as following 1, 2, 3, 4, and 5 min with the energy densities of 60–80, 120–168, 180–240, 252–336, and 300–420 J/cm^2^, respectively. Immediately, 10 μL of each well-containing dilution series were cultured onto BHI agar and the plates were incubated at 37 °C for 24 h. The SSR dose of irradiation time of LED (LED^SSR^) was then determined as mentioned above.

3. *Determination of the antimicrobial effects of ultrasound waves against A. baumannii*

Antimicrobial effect of ultrasound waves against *A. baumannii* was done as described previously [13]. Briefly,100 μL of bacterial cells at the concentration of 1.5 × 10^5^ CFU/mL in the wells of a 96-well microtiter plate were exposed to ultrasound waves at a frequency of 1 MHz for 1 min with five variations of power settings as following 28.7, 36.9, 45.2, 53.4, and 61.6 mW/cm^2^. 10 μL of each well-containing dilution series were immediately cultured onto BHI agar and the agar plates were incubated at 37 °C for 24 h. The SSR dose of ultrasound intensity (US^SSR^) was determined as described above.

4. *Determination of the antimicrobial effects of CurNisNp plus LED irradiation (aPDT) against A. baumannii*

To find out the effect of aPDT based on CurNisNp, 100 μL of CurNisNp^SSR^ was added to 100 μL/well of the *A. baumannii* (1.5 × 10^6^ CFU/mL) in the wells of a 96-well microtiter plate and incubated in the dark room (5 min; 25 °C). The treated bacterial suspensions in microtiter plate wells were immediately exposed with the LED at SSR dose of irradiation time. Finally, the log_10_ CFUs/mL were counted based on the methods mentioned above.

5. *Determination of the antimicrobial effects of CurNisNp plus ultrasound waves (aSDT) against A. baumannii*

aSDT was done as described previously [36]. Briefy, 100 μL of CurNisNp^SSR^ was added to 100 μL/well of the *A. baumannii* (1.5 × 10^6^ CFU/mL) and the microtiter plate was incubated in the dark room (5 min; 25 °C). After that, the sonication was performed with US^SSR^. Eventually, the log_10_ CFUs/mL were counted based on the methods described above.

6. *Determination of the antimicrobial effects of ultrasound waves plus LED irradiation against A. baumannii*

To determine the antimicrobial response to ultrasound waves plus LED, 200 μL of the *A. baumannii* (1.5 × 10^5^ CFU/mL) was added to each well and the microtiter plate was incubated in the dark room for 5 min. Then, the wells were exposed with LED^SSR^ and then US^SSR^. Following growth on the BHI agar plates, the log_10_ CFUs/mL were counted based on the methods mentioned above.

7. *Determination of the antimicrobial effects of CurNisNp plus LED irradiation plus ultrasound waves (aPSDT) against A. baumannii*

100 μL of CurNisNp^SSR^ was added to the 100 μL/well of the *A. baumannii* (1.5 × 10^6^ CFU/mL) in a 96-well microtiter plate. After incubation of microtiter plate for 5 min in the dark room, the bacterial suspension was exposed to LED^SSR^ and then immediately US^SSR^. The treated *A. baumannii* cells were cultured onto BHI agar and the log_10_ CFUs/mL were then counted as described above.

### Experimental design

The test groups consisted of *A. baumannii* subjected to:

A. CurNisNp^SSR^ (CurNisNp at SSR dose)
B. LED^SSR^ (Irradiation time of light source at SSR dose)
C. US^SSR^ (Ultrasound intensity at SSR dose)
D. aPDT^SSR^ (CurNisNp at SSR dose + Irradiation time of light source at SSR dose)
E. aSDT^SSR^ (CurNisNp at SSR dose + Ultrasound intensity at SSR dose)
F. aPSDT^SSR^ (CurNisNp at SSR dose + Irradiation time of light source at SSR dose + Ultrasound intensity at SSR)
G. Control (*A. baumannii* suspension without treatment)
H. Silver sulfadiazine (SSD) (1% w/w as the standard treatment)

### Crystal violet biofilm assay

Static biofilm formation was assayed in the wells of a 96-well microtiter. Briefly, *A. baumannii* cells in BHI broth (total volume 300 μL) at the concentration of 1.5 × 10^8^ CFU/mL were added to wells of a 96-well microtiter plate and incubated at 37 °C for 72 h. After treatment of *A. baumannii* biofilms according to the experimental design described, the biofilms in a 96-well microtiter plate were stained with 0.1% crystal violet for 20 min, dissolved in 95% ethanol, and absorbances were measured spectrophotometrically at 570 nm by a microplate reader (BioTek, Germany). To assess the treatment efficiency of an experimental study on biofilm killing/degradation, the percentage of biofilm killing/degradation was determined as follows:

$$\mathrm{Biofilm}\ \mathrm{killing}/\mathrm{degradation}\%=\frac{\mathrm{OD}\ \mathrm{of}\ \mathrm{untreated}\ \mathrm{slabs}-\mathrm{OD}\ \mathrm{of}\ \mathrm{sample}}{\mathrm{OD}\ \mathrm{of}\ \mathrm{untreated}\ \mathrm{slabs}}\times 100$$

### Measurement of intracellular ROS

The intracellular ROS was estimated using fluorescent2′,7′-dichlorofluorescein diacetate (DCFH-DA) method [55]. Briefly, after centrifugation of the treated *A. baumannii* cell suspensions at 300×g for 30 min, the supernatant was collected and treated with10 μM DCFH-DA for 1 h. The fluorescence intensity of DCF was then quantified with excitation and emission wavelengths of 485 and 530 nm, respectively.

### Mouse model of *A. baumannii*-infected burn wound

All protocols in the animal experiments were approved by the Animal Care Ethics Committee of Tehran University of Medical Sciences (Application no. IR.TUMS.MEDICINE.REC.1399.944). It should be considered that according to the in adherence to international and guidelines for ethical conduct in the care and use of animals [56], in order to observe the ethics of working with experimental animals, after achieving a favorable and acceptable test group in in vitro, the in vivo study was done only in that group.

8 to 12-week-old female BALB/c mice, weighing between 20 g and 25 g (Pasteur Institute, Tehran, Iran) were housed in individual cages under sanitary conditions with a 12-h light/dark cycle and access to the standardized pellet diet and water ad libitum. Each mouse was anesthetized by an intraperitoneal injection of 2% xylazine at a dose of 5 mg/kg and 10% ketamine at a dose of 100 mg/kg. The dorsal skin of the mice was shaved (3 cm × 4 cm) with an electric razor and cleaned with povidone-iodine (10%) and ethanol (70%). As described our previous study [48], a full-thickness, third-degree burn model was prepared. A 50 μL suspension of *A. baumannii* (1.5 × 10^8^ CFU/mL) was dripped into each wound. The wound was fixed with sterile gauze, and the mice were housed in individual cages. As previously reported [47], the duration from inoculation to successful modeling is 24 h. Eight mice were then treated by aPSDT at SSR doses of CurNisNp, irradiation time of LED, and ultrasound intensity after induction of *A. baumannii*, eight mice were treated by SSD, and the other burned mice served as controls (*n* = 4) and received physiological saline instead of any therapeutic treatment. The aPSDT^SSR^ and SSD treatment were done completely in the darkroom and repeated for up to 15 days.

### Evaluation of wound healing potential

The progressive changes of burned area were photographed every 5 days and analyzed using size analysis Software-Image J. The percentage of the wound contracture rate was determined according to the following formula:

$$\%\mathrm{Rate}\ \mathrm{of}\ \mathrm{contracture}=\frac{\mathrm{Specific}\ \mathrm{day}\ \mathrm{wound}\ \mathrm{size}\ }{\mathrm{Initial}\ \mathrm{wound}\ \mathrm{size}} \times 100$$

### Counting of bacteria on wounds

The number of bacteria on the wound surface before (day 0) and on the 1st, 5th, 10th, and 15th days after aPSDT^SSR^ and SSD were measured. At each time point, a part of each ulcer (5 mm in diameter) was cut aseptically using punch biopsy forceps. These specimens were homogenized in 1 mL of sterile saline and the viable bacteria counted as described above.

### Assessment of the virulence-associated genes expression by quantitative real-time PCR

The residual suspensions of treated wound samples were used for RNA extraction. The total RNA extraction was performed using the GeneAll Hybrid-R RNA purification kit (Seoul, Korea) according to the manufacturer’s recommendations. The elimination of genomic DNA and cDNA synthesis were done by RNase-free DNase I treatment and RevertAid First Strand cDNA Synthesis Kit, respectively (both purchased from Thermo Scientific GmbH, Germany). The nucleotide sequences of primers for quantitative real-time PCR as described in Table 2. A housekeeping gene (*16S rRNA*) as the most stable gene was used for the normalization of the reactions. Reaction plates were processed under the following conditions: an initial denaturation of 5 min at 95 °C, followed by 35 cycles of 95 °C for 15 s, annealing for 10 s at 60 °C, and 72 °C for 10 s. Fold differences in RNA expression were calculated by the 2^−∆∆Ct^ method using the Relative Expression Software Tool (REST) 2009 software (version 2.0.13; Qiagen, Valencia, CA, USA), and the changes greater than or equal to two-fold were considered significant [57].

**Table 2:** Primer sequences used in this study

Genes		Sequences (5́ − 3́) ^a^	Amplicon Size (bp) ^b^
***csuE***	F	AGTGTATCGCCGGGTGTTTA	113
	R	AACCCAGGGCTCTCAAAGAA	
***blsA***	F	ACCTTTAACCCGCTTTTGCT	117
	R	TCCCCTATTCACCATTCCAA	
***abaI***	F	TACCCACCACACAACCCTATTT	126
	R	GCGGTTTTGAAAAATCTACGGC	
***16S rRNA***	F	AAAGTTGGTATTCGCAACGG	117
	R	ACCTTTAACCCGCTTTTGCT	

*Abbreviations*: *F* forward primer, *R* reverse primer, and *bp* base pair

^a^ Nucleotides

^b^ Base pair

### Histopathological examinations of burn wound infections

The tissues cross-sections (5–10 mm) from each wound obtained on the 1st, 5th, 10th, and 15th days after aPSDT^SSR^ and SSD were processed for histopathological study. Tissue samples were fixed in 10% buffered formalin in PBS for 72 h. Paraffin-embedded tissue sections of 3–5 μm thick were prepared and stained with hematoxylin-eosin (HE). A general optical microscope (Olympus, Tokyo, Japan) was used to assess the inflammation, fibroblasts, blood vessels, and re-epithelialization.

### Statistical analysis

All experiments were repeated five times. The data of quantitative variables were presented as the median, interquartile range (IQR), and mean ± standard deviation (SD) based on the studies of Pérez-Granda et al. [58] and Alonso et al. [59]. Data were evaluated using one-way analysis of variance (ANOVA) and values *P* < 0.05 were considered statistically significant.

## Data Availability

All data of this manuscript are included in the manuscript. All figures are original images and have been used for the first time in this study. Any additional information required will be provided by communicating with the corresponding author via the official mail: abahador@sina.tums.ac.ir.

## Abbreviations

aPDT: Antimicrobial photodynamic therapy
aSDT: Antimicrobial sonodynamic therapy
aPSDT: Antimicrobial photo-sonodynamic therapy
Cur: Curcumin
Nis: Nisin
ROS: Reactive oxygen species
SSD: Silver sulfadiazine
SSR: Sub-significant reduction
